# Effectiveness of bisphosphonate for alleviating tinnitus associated with otosclerosis: a prospective case–control study

**DOI:** 10.1007/s00405-024-08935-z

**Published:** 2024-09-13

**Authors:** Ayman Fouad, Mahmoud Mandour, Mohamed Osama Tomoum, Reham Mamdouh Lasheen

**Affiliations:** 1 Otolaryngology Department, Faculty of Medicine, Tanta University, El Geish St., Tanta, 31527 Gharbeya Egypt; 2 Otolaryngology Department, Faculty of Medicine, Audiovestibular Unit, Tanta University, Tanta, Egypt

**Keywords:** Otosclerosis, Tinnitus, Bisphosphonate, Tinnitus questionnaire, Risedronate

## Abstract

**Purpose:**

To investigate the short-term efficacy of third-generation bisphosphonate in the management of tinnitus associated with otosclerosis.

**Methods:**

A prospective case–control study included 100 patients with otosclerosis-associated bothersome tinnitus. Patients were assigned to two groups: group A (control): 25 patients who planned to receive only complementary supplements, oral vitamin D plus calcium, and group B (case): 75 patients who planned to receive oral bisphosphonate plus routine vitamin D and calcium supplements. Group B was subdivided into B_1_: 25 patients without any previous intervention, B_2_: 25 patients with persistent tinnitus for more than 6 months after a previous uncomplicated stapedotomy in the same ear, and B_3_: 25 patients with persistent tinnitus for more than 6 months after hearing aid fitting. The outcome was tinnitus assessment both subjectively (tinnitus intensity, frequency, and questionnaire) and objectively (tinnitus intensity and frequency).

**Results:**

The female-to-male ratio was 1.6:1 with ages ranging from 40 to 61 years. The baseline revealed no statistically significant differences between the groups. After 6 months, there were statistically significant differences, both objectively and subjectively. The tinnitus questionnaire median (IQR) for group B was 16 (30), whereas control group A had 52 (24). The tinnitus severity median (IQR) for group B was 20 (30), compared to group A’s 52 (42). After 6 months, 40% of the cases in group B demonstrated complete improvement, compared to 0% in control group A.

**Conclusion:**

We demonstrated significant tinnitus improvement in cases treated with bisphosphonate compared to the control group.

## Introduction

Otosclerosis is a unique temporal bone disease characterized by defective remodeling of the otic capsule in the form of resorption and subsequent disorganized bone regrowth. Patients with otosclerosis typically experience gradual progressive hearing loss and tinnitus [1–4].

Medical therapy, stapedotomy, and hearing aids are the three available forms of treatment of otosclerosis. While surgery is the most effective treatment for stapedial otosclerosis, it may not always be able to fully relieve symptoms, especially tinnitus [2, 3].

Tinnitus is a common symptom in osteosclerotic patients, affecting 68–90% of them. Even after surgery or hearing aids, up to 25% continue to suffer from tinnitus [5, 6].

Since the pathogenesis of otosclerosis entails inflammatory marks, a variety of anti-inflammatory and anti-osteoporotic medications are used to treat it, including sodium fluoride, vitamin D, bioflavonoids, vitamin A, nonsteroidal anti-inflammatory drugs, corticosteroids, immunosuppressive agents, and biological treatments [7].

Bisphosphonates are a class of medications that suppress bone resorption. This activity enables these drugs to be utilized in a variety of disorders, including osteoporosis and Paget’s disease [8].

Bisphosphonate’s efficacy in the treatment of otosclerosis has been extensively examined in terms of hearing stabilization. Since tinnitus has a great impact on the quality of life, we aim to focus on the short-term efficacy of third-generation bisphosphonate “Risedronate” in the management of tinnitus associated with otosclerosis.

## Patients and methods

### Patients

Our study is a prospective case–control study that was conducted in the department of otolaryngology in a tertiary referral center between Jan 2021 and Jan 2024. One hundred patients with otosclerosis-associated bothersome tinnitus lasting more than 6 months were enrolled in our study.

In order to evaluate the efficacy of bisphosphonate therapy, 100 patients were assigned to two groups as follows: group A (control group) included 25 patients planned to receive only complementary supplements, oral vitamin D plus calcium, to rule out the confounding effect of any potential therapeutic impact of these complementary therapies, and group B (case group) included 75 patients planned to receive oral bisphosphonate plus routine vitamin D and calcium supplements. Group B was further subdivided into the following subgroups: group B_1_ included 25 patients without any previous intervention, “no history of stapedotomy or hearing aid”, group B_2_ included 25 patients with persistent tinnitus for more than 6 months after a previous uncomplicated stapedotomy in the same ear (stapedotomy with titanium prosthesis of 0.6 diameter), and group B_3_ included 25 patients with persistent tinnitus for more than 6 months after hearing aid fitting in the same ear.

We excluded patients who suffered from otosclerosis-associated tinnitus for less than 6 months, patients with complicated stapedotomies, patients with bilateral stapedotomies or bilateral hearing aids, patients with contraindications of bisphosphonates therapy (including patients with a history of hypersensitivity to bisphosphonates, hypocalcemia, chronic kidney disease, and esophageal disorders), and patients developed major side effects from bisphosphonate (including femur fracture and jaw osteonecrosis).

### Methods

Patients in group A were treated with vitamin D (1000 international units/day) and calcium (1000 mg/day) supplements. Patients in group B were treated with Risedronate 35 mg orally weekly with the following instructions: to take the medication in the morning with plenty of water, to remain fasting for 30 min, to stay upright for 30 min, and to take vitamin D (1000 international units/day) and calcium (1000 mg/day) supplements.

Demographic data was collected as regards age, sex, duration of tinnitus (measured in groups A and B_1_ between the onset of the tinnitus and the start of the bisphosphonate treatment and measured in groups B_2_ and B_3_ between the surgery or the use of hearing aid and the start of the bisphosphonate treatment, respectively), and side effects from bisphosphonate usage.

### Outcomes

Outcome measures were tinnitus assessment both subjectively and objectively. We made baseline measurements before beginning the bisphosphonate, as well as 3 and 6 months later.

- Subjective intensity: patients were asked to make a self-rating system for the tinnitus loudness, whether mild or severe, and during follow-up, the responses were whether there was no, partial, or complete improvement.
- Subjective frequency: the patients were asked to make a self-rating system for the tinnitus pitch, whether low or high, and during follow-up, the responses were whether there was no change, a change from high to low, or a full resolution.
- Questionnaire: we used the Validated Arabic Version [9] of the Tinnitus Handicap Inventory [10].
- Objective intensity using audiometry: acuphenometry was done, and the patient’s tinnitus was correlated to a certain intensity.
- Objective frequency using audiometry: acuphenometry was done, and the patient’s tinnitus was correlated to a certain frequency.

### Statistical analysis

The data was statistically reported using mean ± standard deviation (m ± SD), median and interquartile range (IQR), or frequencies (number of cases) and percentages when appropriate. All numerical variables were tested for normality, and whenever the variables were normally distributed, the comparison was done using the One-Way ANOVA test. For the not normally distributed numerical variables, the Kruskal–Wallis test and the Mann–Whitney U test were performed. To compare categorical variables, the Chi-square (χ2) test and the Montecarlo test were adopted. The Spearman correlation was applied to correlate the tinnitus intensity with the age, sex and tinnitus duration among subgroups B_1_, B_2_, and B_3_. Simple linear regression and univariate logistic regression were utilized to predict the tinnitus intensity depending on the tinnitus duration among groups B_1_, B_2_, and B_3_. Two-sided p-values less than or equal to 0.05 were deemed statistically significant. All statistical computations were executed on IBM SPSS (Statistical Package for the Social Sciences; IBM Corp, Armonk, NY, USA) release 25 or Microsoft Windows.

## Results

### Demographic data

There was a total of 100 patients who had tinnitus associated with otosclerosis. The overall female-to-male ratio was 1.6:1, with no statistically significant gender difference between the groups (p-value = 0.409). Their age ranged from 40 to 61 years, with a mean of 49.79 ± 6. The groups had no significant age difference (p-value = 0.846) (Table 1).

**Table 1:** Demographic characteristics of the studied patients

Studied variables	Control A	Treatment B_1_	Surgery B_2_	Hearing aid B_3_	Total	P value
Age						
Mean ± SD	48.88 ± 5	49.96 ± 6.6	49.96 ± 5.9	50.36 ± 6.7	49.79 ± 6	0.846^(1)^
Min–Max	41–60	41–60	40–60	40–60	40–60	
Sex						
Male	12	11	7	8	38	0.409^(2)^
	48.0%	44.0%	28.0%	32.0%	38.0%	
Female	13	14	18	17	62	
	52.0%	56.0%	72.0%	68.0%	62.0%	

*Statistically significant at P ≤ 0.05, (1) One way ANOVA test, (2) Chi-square test

### Subjective tinnitus assessment

#### Intensity

The baseline self-rating of tinnitus loudness was mild in 29/100 cases and severe in 71/100 cases, with no statistically significant difference between the groups (p-value = 0.820). After 6 months, there was a statistically significant difference between group B and group A, with a p-value < 0.001. The percentage of cases with partial tinnitus improvement was 50.7% in group B and 12% in group A. The percentage of cases with total tinnitus improvement was 40% in group A and 0% in group B. Within group B, complete improvement of the tinnitus was superior in cases treated with bisphosphonate after stapedotomies (group B_2_) with 64%, followed by cases treated with bisphosphonate after hearing aid usage (group B_3_) with 40%, and finally, cases treated with bisphosphonate only (group B_1_) with 16% (Tables 2, 3).

**Table 2:** Objective and subjective outcomes between case and control groups after 6 months

6th month	Control A	Case B	P value
Subjective intensity			
No improvement			< 0.001^*(1)^
N	22	7	
%	88.0%	9.3%	
Partial improvement			
N	3	38	
%	12.0%	50.7%	
Complete improvement			
N	0	30	
%	0.0%	40.0%	
Subjective frequency			
Complete improvement			< 0.001^*(1)^
N	0	30	
%	0.0%	40.0%	
Low frequency			
N	12	40	
%	48.0%	53.3%	
High frequency			
N	13	5	
%	52.0%	6.7%	
Score of questionnaire			
Median (IQR)	52 (24)	16 (30)	< 0.001^*(2)^
Min–Max	18–64	0–54	
Objective intensity			
Median (IQR)	50 (25)	20 (30)	< 0.001^*(2)^
Min–Max	27–70	0–65	
Objective frequency			
Complete improvement			< 0.001^*(3)^
N	0	30	
%	0.0%	40.0%	
500			
N	6	21	
%	24.0%	28.0%	
1000			
N	5	17	
%	20.0%	22.7%	
4000			
N	9	4	
%	36.0%	5.3%	
6000			
N	4	3	
%	16.0%	4.0%	
8000			
N	1	0	
%	4.0%	0.0%	

*IQR* interquartile range

*Statistically significant at P ≤ 0.05, (1) Chi-square test, (2) Montecarlo test, (3) Kruskal Wallis test

**Table 3:** Subjective tinnitus assessment at 0, 3, and 6 months

Time in months	Degree	Control A	Treatment B_1_	Surgery B_2_	Hearing aid B_3_	P value
Subjective intensity						
0	Mild					
	N	7	6	7	9	0.820^(1)^
	%	28.0%	24.0%	28.0%	36.0%	
	Severe					
	N	18	19	18	16	
	%	72.0%	76.0%	72.0%	64.0%	
3	No improvement					
	N	22	12	1	1	< 0.001^*(2)^
	%	88.0%	48.0%	4.0%	4.0%	
	Partial improvement					
	N	3	13	11	18	
	%	12.0%	52.0%	44.0%	72.0%	
	Complete improvement					
	N	0	0	13	6	
	%	0.0%	0.0%	52.0%	24.0%	
6	No improvement					
	N	22	7	0	0	< 0.001^*(2)^
	%	88.0%	28.0%	0.0%	0.0%	
	Partial improvement					
	N	3	14	9	15	
	%	12.0%	56.0%	36.0%	60.0%	
	Complete improvement					
	N	0	4	16	10	
	%	0.0%	16.0%	64.0%	40.0%	
Subjective frequency						
0	Low frequency					
	N	8	8	7	8	0.987^(1)^
	%	32.0%	32.0%	28.0%	32.0%	
	High frequency					
	N	17	17	18	17	
	%	68.0%	68.0%	72.0%	68.0%	
3	Complete improvement					
	N	0	0	13	6	< 0.001^*(2)^
	%	0.0%	0.0%	52.0%	24.0%	
	Low frequency					
	N	8	17	11	12	
	%	32.0%	68.0%	44.0%	48.0%	
	High frequency					
	N	17	8	1	7	
	%	68.0%	32.0%	4.0%	28.0%	
6	Complete improvement					
	N	0	4	16	10	< 0.001^*(2)^
	%	0.0%	16.0%	64.0%	40.0%	
	Low frequency					
	N	12	17	9	14	
	%	48.0%	68.0%	36.0%	56.0%	
	High frequency					
	N	13	4	0	1	
	%	52.0%	16.0%	0.0%	4.0%	
Score of questionnaire						
0	Median (IQR)	52 (29)	56 (27)	54 (27)	56 (41)	0.587^(3)^
	Min–Max	18–64	16–66	18–64	16–66	
3	Median (IQR)	50 (23)	44 (32)	0 (51)	36 (40)	
	Min–Max	18–62	16–64	0–60	0–60	0.002^*(3)^
	Pairwise comparison	P1 > 0.999, P2 = 0.002*, P3 = 0.090, P4 = 0.037*, P5 = 0.614, P6 > 0.999				
6	Median (IQR)	52 (24)	20 (15)	0 (16)	16 (38)	< 0.00^*(3)^
	Min–Max	18–64	0–54	0–52	0–50	
	Pairwise comparison	P1 = 0.002*, P2 < 0.001*, P3 < 0.001*, P4 = 0.070, P5 > 0.999, P6 = 0.823				

P1(A–B_1_), P2(A–B_2_), P3(A–B_3_), P4(B_1_–B_2_), P5(B_1_–B_3_), P6(B_2_–B_3_)

*IQR* interquartile range

*Statistically significant at P ≤ 0.05, (1) Chi-square test, (2) Montecarlo test, (3) Kruskal Wallis test

#### Frequency

The baseline self-rating of tinnitus pitch was low in 31/100 cases and high in 69/100 cases, with no statistically significant pitch difference between the groups (p-value = 0.987). Over time, some cases revealed complete tinnitus improvement, and others demonstrated a pitch change from high to low. For example, at baseline in the control group A, 32% had low-pitch tinnitus and 68% had high-pitch tinnitus, and after 6 months, 0% demonstrated complete resolution, 48% had low-pitch tinnitus, and 52% had high-pitch tinnitus. On the contrary, at baseline in group B, 31% had low-pitch tinnitus and 69% had high-pitch tinnitus, and after 6 months, 40% demonstrated complete resolution, 53% had low-pitch tinnitus, and 7% had high-pitch tinnitus. Within group B, group B_2_ was better, followed by group B_3_, and then group B_1_ (Tables 2, 3).

#### Tinnitus questionnaire

The baseline tinnitus questionnaire score, reflected by the median (IQR), was 54 (27), with no statistically significant difference between the groups (p-value = 0.587). After 6 months, the median (IQR) for group B was 16 (30), whereas control group A had 52 (24). The difference was statistically significant (p-value < 0.001). Within group B, the Pairwise comparison test demonstrated a difference between the three subgroups, however it was statistically insignificant (Tables 2, 3).

### Objective tinnitus assessment

#### Intensity

The baseline objective tinnitus intensity was 60 (25), given as a median (IQR), with no statistically significant difference between the groups (p = 0.830). After 6 months, the median (IQR) for group B was 20 (30), compared to the control group A of 52 (42). The difference was statistically significant (p-value < 0.001). Within group B, group B_2_ showed greater improvement in tinnitus compared to groups B_1_ and B_3_, with p-values < 0.001 and = 0.007, respectively. However, no significant difference was seen between groups B_1_ and B_3_ (p-value > 0.999) (Tables 2, 4).

**Table 4:** Objective Tinnitus assessment at 0, 3, and 6 months

	Control A	Treatment B_1_	Surgery B_2_	Hearing aid B_3_	P value
Objective intensity					
0					
Median (IQR)	55 (25)	60 (25)	60 (30)	55 (20)	0.830^(1)^
Min–Max	30–70	30–80	30–75	30–75	
3					
Median (IQR)	55 (23)	50 (25)	0 (35)	40 (38)	< 0.001^*(1)^
Min–Max	30–70	30–75	0–50	0–55	
Pairwise comparison	P1 > 0.999, P2 < 0.001*, P3 = 0.003*, P4 < 0.001*, P5 = 0.296, P6 = 0.027*				
6					
Median (IQR)	50 (25)	35 (28)	0 (20)	20 (30)	< 0.001^*(1)^
Min–Max	25–70	0–95	0–40	0–40	
Pairwise comparison	P1 = 0.301, P2 < 0.001*, P3 < 0.001*, P4 < 0.001*, P5 > 0.999, P6 = 0.007*				
Objective frequency					
0					
500					
N	2	2	1	2	0.429^(2)^
%	8.0%	8.0%	4.0%	8.0%	
1000					
N	6	6	6	6	
%	24.0%	24.0%	24.0%	24.0%	
4000					
N	4	6	4	3	
%	16.0%	24.0%	16.0%	12.0%	
6000					
N	6	11	8	8	
%	24.0%	44.0%	32.0%	32.0%	
8000					
N	7	0	6	6	
%	28.0%	0.0%	24.0%	24.0%	
3					
Complete improvement					
N	0	0	13	6	< 0.001*^(2)^
%	0.0%	0.0%	52.0%	24.0%	
500					
N	5	9	3	3	
%	20.0%	36.0%	12.0%	12.0%	
1000					
N	3	8	8	8	
%	12.0%	32.0%	32.0%	32.0%	
4000					
N	8	3	1	3	
%	32.0%	12.0%	4.0%	12.0%	
6000					
N	7	5	0	5	
%	28.0%	20.0%	0.0%	20.0%	
8000					
N	2	0	0	0	
%	8.0%	0.0%	0.0%	0.0%	
6					
Complete improvement					
N	0	4	16	10	< 0.001*^(2)^
%	0.0%	16.0%	64.0%	40.0%	
500					
N	6	10	5	6	
%	24.0%	40.0%	20.0%	24.0%	
1000					
N	5	7	4	6	
%	20.0%	28.0%	16.0%	24.0%	
4000					
N	9	1	0	3	
%	36.0%	4.0%	0.0%	12.0%	
6000					
N	4	3	0	0	
%	16.0%	12.0%	0.0%	0.0%	
8000					
N	1	0	0	0	
%	4.0%	0.0%	0.0%	0.0%	

P1(A–B_1_), P2(A–B_2_), P3(A–B_3_), P4(B_1_–B_2_), P5(B_1_–B_3_), P6(B_2_–B_3_)

*IQR* interquartile range

*Statistically significant at P ≤ 0.05, (1) Chi-square test, (2) Montecarlo test

#### Frequency

The baseline objective tinnitus frequencies were 7, 24, 17, 33, and 19% at 500, 1000, 2000, 4000, and 8000 Hz, respectively. The groups showed no statistically significant difference (p = 0.429). After 6 months, 40% of the cases in group B demonstrated complete improvement, compared to 0% in control group A. Those who did not improve completely after 6 months in group B experienced a frequency shift from high to low (Tables 2, 4).

### Correlations

**Fig. 1:**
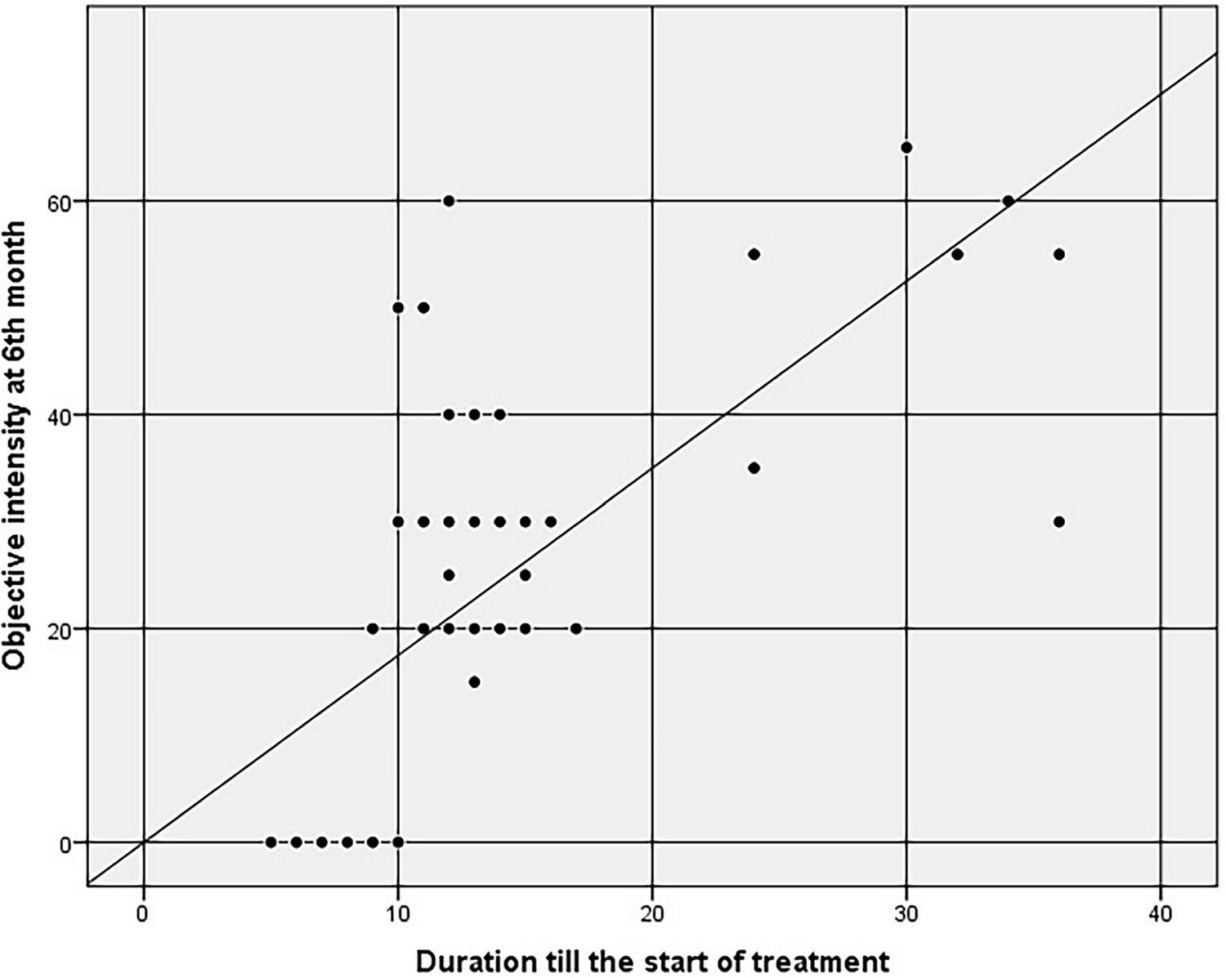
Correlation between objective tinnitus intensity after 6 months and the duration between tinnitus till the start of the bisphosphonate therapy in group B

In group B, a correlation was conducted between tinnitus intensity improvement after 6 months and age, sex, and tinnitus duration. Regarding age and sex, the correlation was determined to be statistically insignificant (p = 0.251 and 0.145, respectively). Concerning tinnitus duration, the correlation was deemed statistically significant (p-value < 0.001). Simple linear regression was utilized to predict the objective tinnitus intensity based on the tinnitus duration (Fig. 1). We demonstrated that the longer the tinnitus duration, the higher the objective tinnitus intensity score with the equation [Intensity = − 4.318 + (2.011 × duration in months)]. For example, patients who began bisphosphonate treatment after complaining of tinnitus for 6 and 12 months had a higher intensity score of 8 and 20 dB, respectively. Univariate logistic regression was utilized to predict the subjective tinnitus intensity based on the tinnitus duration. We revealed that for every 1-month increase in tinnitus duration, the odds of complete tinnitus improvement decrease by 7.6%.

### Bisphosphonate tolerance

In control group A, 3/25 cases experienced gastric upset symptoms and were treated with a proton pump inhibitor (PPI). In group B, 10/75 cases developed gastric upset symptoms and were treated with a PPI. There was no statistical significance between the two groups (p-value = 0.864).

## Discussion

Our objective in this prospective case–control study was to evaluate the effect of bisphosphonates in the management of patients with otosclerosis-associated tinnitus. In comparison to the control group, we demonstrated a noteworthy tinnitus improvement in cases treated with bisphosphonates. To the best of our knowledge, this is the first work to focus on tinnitus management in osteosclerotic patients using bisphosphonates.

### Tinnitus and otosclerosis

In our study, the female-to-male ratio was 1.6. Previous literature found that otosclerosis is more common in females [2, 3], tinnitus in general is more common in females [11], and tinnitus associated with otosclerosis is more common in females [11, 12]. The mean age was 49 years. No sex or age difference was reported between groups.

Tinnitus presents in 70–85% of cases with different auditory system disorders [13]. Tinnitus’s exact pathophysiology is still being investigated. Some theories stated that an efferent auditory channel controls the organ of Corti’s outer hair cells. When the auditory stimulus is attenuated, the brain compensates by increasing the sensitivity of the hair cells, which generates a phantom tinnitus perception [14, 15].

Tinnitus is a troublesome symptom of otosclerosis, and it was reported in 65–92% of cases [16–18]. Even after a successful uncomplicated stapedotomy, tinnitus persists in 20–50% of cases [16].

The exact pathophysiology of tinnitus in otosclerosis is still unclear. However, there are many theories that explain. Patients with fixed footplates have less perilymph vibration, resulting in less afferent stimulation of the central auditory system. The decreased afferent stimulation decreases the suppression of the efferent pathways on the Corti’s organ and hence tinnitus production [14].

The frequency of tinnitus usually correlates with the frequency of hearing loss. A decrease in cochlear input at a certain frequency result in over-signaling of the same frequencies in the central pathways. For this reason, the pitch of the tinnitus is perceived similar to the hearing loss frequencies [19, 20].

Chronic tinnitus is defined as tinnitus lasting more than 3 months. So, in our study, we included cases who experienced tinnitus associated with otosclerosis for at least 6 months and were followed up for at least 6 months after treatment.

### Medical treatment for otosclerosis

Otosclerosis is a unique otic bone disease for which there are numerous medical therapeutic options available, particularly sodium fluoride (NaF) and bisphosphonates [21].

NaF is an effective medication for treating active otosclerosis, in which NaF can stabilize progressive hearing loss. Bisphosphonates can replace NaF if the patient is intolerant of it, or developed a contraindication to NaF, or as an adjuvant to NaF. Bisphosphonates can be used as an adjuvant to a stapedotomy, a hearing aid, or as a primary treatment [21].

Bisphosphonate is a well-established treatment for a variety of metabolic bone disorders, such as osteoporosis and Paget diseases [8]. Bisphosphonate acts by interfering with osteoclast and triggering their apoptosis, hence suppressing bone resorption. Their mechanism of action in otospongiosis is not well defined. Otosclerosis-induced hearing loss results from affection of the endosteal layer of the otic bone, which leads to spiral ligament hyalinization [22]. Perhaps bisphosphonates slow down hyalinization and thereby halt the progression of hearing loss in these patients. Bisphosphonate may inhibit the release of tumor necrosis factor (TNF) alpha, which is toxic to hair cells [23]. Decreasing the perilymph TNF-alpha levels should have a stabilizing effect on hair cells and hearing [24]. Bisphosphonates may directly affect the spiral ganglion population and improve their survival [25].

Some authors found that the required dose of bisphosphonate for treating otosclerosis is double that of osteoporosis. This higher dose is justified by the fact that normal otic capsule contains no osteoclasts, as opposed to the bone subjected to osteoporosis [26].

Bisphosphonates are classified according to their generation (old or new), route of administration (oral or intravenous), and dose frequency (daily, monthly, or annually).

Bisphosphonates reduce the production of osteoclasts. Older generations, as etidronate, have an undesirable effect by inhibiting osteoblastic activity. As a result, these generations cannot be exploited continuously. Newer generations, by adding a nitrogen molecule, act more selectively on osteoclasts while sparing osteoblasts and could be utilized on a continuing basis. Kennedy [27] used the old generation, etidronate, Oliveira [28] used the newer generation, alendronate. There is no study to compare the differences between different forms of bisphosphonates. However, in general, newer generations are more effective, potent, safer, and used in smaller doses. Some authors advocate alternative use of a new generation (risedronate) together with an older generation (etidronate) to exert the maximum benefit on the inner ear [26, 29].

Examples of oral third-generation bisphosphonates are alendronate and risedronate, which are used at daily doses of 10 and 5 mg or weekly doses of 70 and 35 mg, respectively [21].

Examples of intravenous bisphosphonates are clodronate, pamidronate, and zoledronate, which are used in doses of 1500 mg (monthly), 90 mg (monthly), or 4 mg (yearly), respectively [30]. Zoledronate possesses a higher bony affinity, a thousand times greater potency compared to etidronate, a favorable tolerability profile with a single intravenous dosage per year, and a prolonged effect of up to 5 years [31, 32].

We chose risedronate, a third-generation bisphosphonate, for our trial because of its potency and tolerability compared to other oral bisphosphonates [33]. We adopted a weekly 35 mg treatment instead of a daily 5 mg protocol to ensure patient adherence to therapy. Bisphosphonates can cause hypocalcemia, particularly in vitamin D-deficient people; therefore, vitamin D and calcium supplements are recommended. Because vitamin D may have therapeutic effects on otosclerosis, we provided the control group both vitamin D and calcium supplements to rule out any confounding effects of these agents [34–36].

Bisphosphonates causes some side effects, some of which are serious. Most commonly, with oral administration, patients may experience mild esophageal irritation, gastritis, or dysphagia, whereas intravenous administration may induce a mild acute inflammatory response such as fever, myalgia, or arthralgia. Hypocalcemia is reported, for which calcium and vitamin D are used as supplements. Rarely, nephrotoxicity, mandibular osteonecrosis, atypical femur fractures, and orbital inflammation have been documented. Previous trials conducted over a five- to ten-year period revealed no serious issues [31, 37].

In our study, a few occurrences of gastric upset resulted from bisphosphonates, which can be adequately controlled with PPI.

However, given these possible hazards, a local application of bisphosphonates is being investigated to limit systemic side effects. Kang et al. [38] described the local application of bisphosphonates at the round window membrane in guinea pigs. Another study [39] applied bisphosphonates locally at the oval window in fresh cadaveric human temporal bone. Further investigation is still needed to determine the safety, optimal dose, and possibility of local application of bisphosphonates in human ears.

Numerous studies have been conducted to determine the usefulness of bisphosphonates in treating otosclerosis. Some studies focused on audiological data and demonstrated not just hearing stabilization but also hearing improvement [24, 29, 37, 40]. Other studies employed computed tomography (CT) [37, 41] or magnetic resonance imaging (MRI) [42] to show evidence of remineralization at the otic capsule. Both audiological and radiological data require longer time to pick up subtle changes [29].

However, few studies have specifically assessed the effect of bisphosphonate on otosclerosis-associated tinnitus. To best of our knowledge we are the first to report.

### Tinnitus evaluation

Tinnitus is a subjective symptom that can present in numerous forms and can vary throughout time in the same patient. As a result, its evaluation and reporting are challenging, contentious, and controversial. Tinnitus has a great impact on the quality of life. In the English literature, we found no other studies that specifically reported tinnitus outcomes after bisphosphonates therapy for otosclerosis to compare with.

In the present study, we performed both objective and subjective evaluations to confirm the therapeutic efficacy of bisphosphonates, which were highly effective in alleviating tinnitus. Subjectively, in the case group, 40% of participants experienced complete tinnitus remission after 6 months, with a significant improvement in the tinnitus questionnaire score compared to 0% in the control group. The treatment group showed a significant improvement in median tinnitus intensity when compared to the control group.

We employed THI as a method to evaluate tinnitus. The median score was 54, which is categorized as a moderate tinnitus handicap. In the literature, a moderate-to-severe tinnitus handicap score was reported in 40–50% of cases [12].

There are other methods used to evaluate tinnitus: Ayache et al. [43] adopted a self-created 4-point scale (unbearable, irritating, bothersome, and slightly bothersome). In another publication, the visual analog score (VAS) was utilized to quantify tinnitus-related distress [17, 18, 43].

Tinnitus associated with otosclerosis may be of high [43] or low frequency [11]. We found that bisphosphonates are more effective at higher frequencies. This is consistent with prior research demonstrating that sodium fluoride and bisphosphonates predominantly improve high-frequency hearing loss [21, 29, 37].

Within group B, we found that subgroup B_2_ ‘who underwent previous stapedotomies’ had considerably better tinnitus improvement than subgroup B_1_ ‘who received therapy alone’ and subgroup B_3_ ‘who utilized hearing aids’. Perhaps there is a synergistic effect between surgical treatments that act mostly on low frequencies and bisphosphonates, which act predominantly on high frequencies.

This is supported by previous research showing that the low-frequency tinnitus is more likely to improve after surgery, and high pitched-tinnitus may persist even after complete air–bone gap closure. Many researchers found that when the vibration of the perilymph is surgically restored and the hearing loss is alleviated, the low-pitched tinnitus largely resolves [5, 6, 17, 44].

So, all otosclerosis patients with tinnitus should be evaluated concerning their tinnitus frequencies pre-operatively. Patients with high-frequency tinnitus should be counseled that their tinnitus may persist even after successful hearing improvement by surgery.

We determined that the longer the tinnitus lasted before starting treatment, the worse the prognosis. As a result, we recommended commencing bisphosphonates as soon as feasible.

### Strength and limitations

Strength of our study included (1) we focused on tinnitus evaluation pre- and post-treatment, (2) we investigated the effect of bisphosphates on a mosaic group (primary otosclerosis, otosclerosis with stapedotomy, and otosclerosis with hearing aids), and (3) we evaluated the tinnitus using both subjective (frequency, intensity, and THI) and objective (frequency and intensity) methods, allowing for future comparisons with other studies.

Limitations of our study included (1) we concentrated solely on tinnitus and did not examine any audiological findings, this is because there have been numerous previous studies that focus on the hearing effects of bisphosphonates and 6 months are insufficient to capture subtle changes in audiology, (2) we followed up on cases for 6 months only, which is relatively short, and (3) there were a restricted number of patients treated in our study.

## Conclusion

Compared to the control group, we demonstrated significant tinnitus improvement in cases treated with bisphosphonates both objectively and subjectively. We found a synergistic effect between surgical treatments that act mostly on low frequencies and bisphosphonates which act predominantly on high frequencies. The earlier the start in bisphosphonates, the better the prognosis. The short-term treatment with bisphosphonates is well tolerated without significant side effects.

## Data Availability

Data is available upon reasonable request due to privacy/ethical restrictions.
